# European clinical guidelines for Tourette syndrome and other tic disorders—version 2.0. Part IV: deep brain stimulation

**DOI:** 10.1007/s00787-021-01881-9

**Published:** 2021-10-04

**Authors:** Natalia Szejko, Yulia Worbe, Andreas Hartmann, Veerle Visser-Vandewalle, Linda Ackermans, Christos Ganos, Mauro Porta, Albert F. G. Leentjens, Jan-Hinnerk Mehrkens, Daniel Huys, Juan Carlos Baldermann, Jens Kuhn, Carine Karachi, Cécile Delorme, Thomas Foltynie, Andrea E. Cavanna, Danielle Cath, Kirsten Müller-Vahl

**Affiliations:** 1 Department of Neurology, Medical University of Warsaw, Banacha 1a, 02-091 Warsaw, Poland; 2 Department of Bioethics, Medical University of Warsaw, Banacha 1a, 02-091 Warsaw, Poland; 3 Department of Neurology, Yale School of Medicine, Yale University, New Haven, USA; 4 Department on Neurophysiology, Saint Antoine Hospital, Sorbonne Université, Paris, France; 5 National Reference Center for Tourette Disorder, Pitié Salpetiere Hospital, Paris, France; 6 Department of Stereotactic and Functional Neurosurgery, Faculty of Medicine and University Hospital Cologne, University of Cologne, Cologne, Germany; 7 Department of Neurosurgery, Maastricht University Medical Center, Maastricht, The Netherlands; 8 Department of Neurology, Charité Universitätsmedizin Berlin, Berlin, Germany; 9 Department of Neurosurgery and Neurology, IRCCS Instituto Ortopedico Galeazzi, Milan, Italy; 10 Department of Psychiatry, Maastricht University Medical Center, Maastricht, The Netherlands; 11 Department of Neurosurgery, Ludwig-Maximilians-University of Munich, Munich, Germany; 12 Department of Psychiatry and Psychotherapy, University of Cologne, Cologne, Germany; 13 Department of Psychiatry, Psychotherapy, and Psychosomatics, Johanniter Hospital Oberhausen, Oberhausen, Germany; 14 Department of Neurosurgery, Pitié-Salpetriere Hospital, Sorbonne Université, Paris, France; 15 Department of Neurology, Pitié-Salpetriere Hospital, Sorbonne Université, Paris, France; 16 Department of Clinical and Movement Neurosciences, UCL Queen Square Institute of Neurology, London, UK; 17 Institute of Clinical Sciences, University of Birmingham, Birmingham, UK; 18 Department of Specialist Trainings, GGZ Drenthe Mental Health Institution, Assen, The Netherlands; 19 Department of Psychiatry, University Medical Center Groningen, Rijks University Groningen, Groningen, The Netherlands; 20 Clinic of Psychiatry, Social Psychiatry and Psychotherapy, Hannover Medical School, Hannover, Germany

**Keywords:** Tics, Tourette syndrome, Deep brain stimulation, Treatment, Guidelines, European Society for the Study of Tourette Syndrome (ESSTS)

## Abstract

In 2011 the European Society for the Study of Tourette Syndrome (ESSTS) published its first European clinical guidelines for the treatment of Tourette Syndrome (TS) with part IV on deep brain stimulation (DBS). Here, we present a revised version of these guidelines with updated recommendations based on the current literature covering the last decade as well as a survey among ESSTS experts. Currently, data from the International Tourette DBS Registry and Database, two meta-analyses, and eight randomized controlled trials (RCTs) are available. Interpretation of outcomes is limited by small sample sizes and short follow-up periods. Compared to open uncontrolled case studies, RCTs report less favorable outcomes with conflicting results. This could be related to several different aspects including methodological issues, but also substantial placebo effects. These guidelines, therefore, not only present currently available data from open and controlled studies, but also include expert knowledge. Although the overall database has increased in size since 2011, definite conclusions regarding the efficacy and tolerability of DBS in TS are still open to debate. Therefore, we continue to consider DBS for TS as an experimental treatment that should be used only in carefully selected, severely affected and otherwise treatment-resistant patients.

## Introduction

Tourette syndrome (TS) is a chronic motor and vocal tic disorder. The prevalence of TS in general population is estimated at 0.3–1% [1–3]. After the onset, usually at the age of about 4–6 years, tics tend to have a waxing and waning course over the years and generally reach a maximum severity around 12 years [4]. In the vast majority of patients, thereafter tics decrease during adolescence or early adulthood and overall have a favorable prognosis. In those patients suffering from disabling tics, behavioral and/or pharmacotherapy is recommended as first line treatments [5–8].

A minority of patients experiences a persistent course and does not benefit from well-established treatments and/or experience serious side effects such as significant weight gain, hyperprolactinemia, somnolence and tiredness [9]. To date, there is no generally established definition available for “treatment refractoriness” and exact number of “treatment-refractory” patients is unknown [10–12]. However, there is broad agreement that various types of treatments in adequate doses, frequency, and duration should have been used before classifying a patient as otherwise “treatment-refractory”. Surgical treatment with deep brain stimulation (DBS) should be taken into consideration only in patients with severe and otherwise treatment-refractory tics. In most of these severely affected patients, psychiatric comorbidity such as attention-deficit/hyperactivity disorder (ADHD), obsessive–compulsive symptoms (OCS) or disorder (OCD), depression, anxiety, and self-injurious behavior (SIB) is present that often impairs patients’ quality of life more than the tics [13–16]. Thus, before considering DBS for tics in TS, the best possible treatment of psychiatric symptoms—notably obsessions, compulsions and depression—should have been carried out [17, 18].

In TS, ablative surgery with prefrontal lobotomy was first performed in 1955 [19]. The first surgical treatment using the thalamus as a target was undertaken in 1970 by Hassler and Dieckmann [20], who performed thalamotomy in the centromedial–parafascicular complex (CM–Pf) and nucleus ventro-oralis internus (Voi) in three patients, which resulted in a tic reduction of 70–100% [21]. Subsequently, Babel et al. [22] reported tic reduction in 14 of 17 patients after ventriculography-based stereotactic zona incerta (ZI) and ventrolateral/lamella medialis thalamotomy (VL/LM). However, in a large number of patients postoperative complications occurred including (transient or permanent) cerebellar ataxia, dysarthria, dystonia, and hemiballism. Finally, in 1999 [23] thalamic deep brain stimulation (DBS) was performed for the first time in three otherwise treatment-refractory adult patients with TS. Subsequently, several other targets have been suggested.

In 2011, the European Society for the Study of Tourette Syndrome (ESSTS) published its first European clinical guidelines on the treatment of TS with part IV on DBS [18]. Here, we provide our updated guidelines with recommendations for the clinical practice of DBS in TS based not only on the evidence obtained over the past decade, but also on expert knowledge including results from a survey among a large number of ESSTS experts.

## Methods

For the current version of part IV of the European clinical guidelines, a literature search was carried out. The aim was to identify relevant research on efficacy and safety of DBS for TS published between January 2011 and August 2021. Our systematic approach was based on the search in PubMed, Ovid, Web of Science, Embase, and APA Psych Info conducted on March 2020 and again on August 27, 2021. We searched for articles reporting about DBS in TS using the search terms “tics” AND/OR “Tourette” AND/OR “deep brain stimulation” AND/OR “DBS”. Reviews and meta-analyses in the area were further searched for relevant citations. In addition, the reference lists of the articles identified were reviewed for additional studies. In addition to the studies identified through systematic review, to make the publication list as comprehensive as possible, studies still in press and not officially published were added by the authors (i.e. through precedent knowledge about relevant publications). The methodology of the ESSTS survey is presented in a summary paper in the current issue of this journal [24].

## Review of the literature (based on search of studies on DBS between 1970 and August 2021)

*Preliminary note: The available evidence for DBS in TS is still very limited. Available evidence is based on few small randomized controlled trials (RCTs), open uncontrolled case reports and case series, registries, and meta-analyses. It is important to note that data largely overlap and results from one single patient may be included in more than one report. To give the best possible overview and comparability, we report data depending on the study design. For more detailed results we refer to the original publications.*

When the first version of the ESSTS DBS guidelines was published in 2011 [18], only 3 RCTs were available, each including 1 to 5 patients [25–27] with altogether nine patients. In addition, open and uncontrolled reports covering a total of 63 patients were published, which reported beneficial outcomes with moderate to marked tic improvement in the vast majority of patients (59/63 patients). Since this review, five subsequent RCTs have been published, resulting in a total of 62 patients in all RCTs. Thus, until today there is still only a total of eight RCTs available including a maximum of 17 patients [25–32]. A summary of these studies is presented in Table 1. In addition, data from the International Deep Brain Stimulation Database and Registry including 185 patients (published in 2018 [33]), one retrospective analysis on long-term follow-up in 110 patients (published in 2019 [34]), and two meta-analyses (published in 2016 and 2018) including 58 [35] and 156 [36] patients were published. In addition, data from several further open uncontrolled studies and case series were published including 2 to 123 subjects as well as more than 100 single case reports (for summary see Table 2).

**Table 1:** RCTs using DBS in TS (in chronological order)

Authors	Numbers of patients*	Target	Duration (months)		Mean tic improvement (according to YGTSS)**	
			Double-blind controlled study part	Open uncontrolled study part	Double-blind controlled study part	Open uncontrolled study part
Houeto et al. [25]	1/1	CM-Pf, internal part of the GPi	11	24	Tic reduction of 59–70% depending on target Mean tic reduction of 41.25 according to YGTSS-TTS	“Improvement” (no data shown)
Maciunas et al. [26]	5/5	Bilateral thalamic stimulation	1	3	Mean tic reduction of 2.4 according to YGTSS-TTS	Improvement in 3/5 patients Mean tic reduction of 9.0 according to the YGTSS-TTS at the 3-Month follow-up Mean overall reduction of 38.8 (44%) according to YGTSS-GS at 3 months compared with the preoperative score
Welter et al. [27]	3/3	GPi and thalamic stimulation	8	20–60	Tic improvement ranging from 30 to 96% according to YGTSS-TTS	Tic improvement ranging from 74 to 82% according to YGTSS-TTS
Ackermans et al. [31]	6/6	Thalamic stimulation	6	12	Significant improvement (*p* = 0.046) of 37% according to YGTSS-TTS Mean YGTSS-TTS improvement of 15.5	Significant improvement (*p* = 0.028) of 49% according to YGTSS-TTS Mean YGTSS-TTS improvement of 20.7
Kefalopoulou et al. [30]	13/15	GPi	6	8–36	Significant tic improvement according to YGTSS-TTS (*p* = 0.048) Mean improvement in YGTSS-TTS of 12.4	Significant improvement according to YGTSS-TTS (*p* < 0.0001) Mean improvement in YGTSS-TTS of 36.3
Welter et al. [29]	16/16	GPi	3	6	No significant improvement (*p* = 0.39) Mean reduction of 1.2 according to YGTSS-TTS	Significant improvement (no p shown) Mean reduction of 30.3 according to YGTSS-TTS
Müller-Vahl et al. [28]	10/10	GPi vs thalamic stimulation	9	6–89.9	Significant tic reduction at group level, after GPi—but not thalamic—DBS compared to baseline No effects on premonitory urges and psychiatric comorbidities Inconsistent or negative findings when comparing targets directly	At group level, no improvement of tics, comorbidities, and quality of life Single patients benefitted continuously from thalamic DBS At last follow-up 89.9 months (mean) after surgery, 50% of patients had discontinued DBS
Baldermann et al. [32]	8/8	Thalamic stimulation	48 h of ON followed by 48 h sham stimulation at 6 and 12 months	12	Significant tic reduction according YGTSS (*p* = 0.001) YGTSS tic scores were significantly decreased with active stimulation by 26% compared to discontinued stimulation after 6 months and by 44% after 12 months	YGTSS tic scores decreased significantly from baseline to 6 months (*p* < 0.001) and twelve months (*p* = 0.001) but not from 6 months to 12 months (*p* = 1.0)

*RCTs* randomized controlled trials, *YGTSS* Yale Global Tics Severity Scale, *YGTSS-TTS* Total Tic Score of the YGTSS, *DBS* Deep Brain Stimulation, *TS* Tourette syndrome, *GPi* Globus pallidus internus, *CM-Pf* the centromedian–parafascicular nuclei complex of thalamus

*The first number refers to the number of patients included in the double-blind controlled study phase, the second number refers to those in the open uncontrolled study phase **when no mean improvement according to YGTSS was shown, we present the descriptive results

**Table 2:** Published case reports and other open uncontrolled studies using DBS in patients with TS

Single case reports		
N of studies	*N* (patients)	References
35	1	[23, 37–70]

Case series		
N of studies	*N* of patients (range)	References
25	2–55	[37, 71, 71–94]

Open uncontrolled studies		
N of studies	*N* of patients (range)	References
28	1–123	[34, 95, 95–122]

*DBS* deep brain stimulation, *TS* Tourette syndrome

### Randomized controlled trials

To date, since 1999, 8 RCTs examining efficacy and safety of DBS in TS have been published with a total of 62 patients (for further details including target, treatment duration, effects on tics and comorbidities see Table 1). However, results are still limited by small sample sizes (ranging from 1 to 17 patients). The first trial by Houeto et al. [25] included only one patient who was treated with bilateral high frequency stimulation of the centre median nucleus/parafascicular complex (CM–Pf), the internal part of the globus pallidus (GPi), or both. Stimulation of either target resulted in tic improvement of 70%, markedly ameliorated coprolalia, and eliminated SIB. In another double-blind crossover trial of bilateral thalamic DBS in five adults with TS [26] a statistically significant (*p* < 0.03) reduction in the modified Rush Video-Based Rating Scale was identified and motor and vocal tics significantly improved according to the Yale Global Tic Severity Scale Total Tic Score (YGTSS-TTS). Welter et al. [27] included three patients with severe, medically refractory TS who received bilateral stimulation in the CM-Pf and the GPi. Both interventions resulted in significant improvement of tics ranging from 30 to 96%. The two largest RCTs (both using GPi DBS) included 15 [30] and 17 [29] patients respectively, and demonstrated overall tic improvement during the blinded study phase of 15.3% (*p* = 0.048) on the YGTSS-TTS in one study [29], but no improvement in the other (median tic reduction of 1.1%, *p* = 0.39) [30]. However, in both studies a significant improvement was reported at the end of the open-label phase after several months (6–48 months) with a tic reduction of 40.1% and 69.5%, respectively [29, 30, 95]. In the third largest RCT [28] (comparing efficacy and safety of bilateral DBS of CM-Voi versus posteroventral lateral pvl) Gpi versus sham stimulation), 10 patients were included and GPi DBS (*p* = 0.05)—but not thalamic DBS (*p* = 0.18)—resulted in a significant tic reduction compared to baseline, but had no effect on premonitory urges and psychiatric comorbidities. Direct comparisons of both targets to sham stimulation resulted in inconsistent or negative findings. During follow-up, at group level, no improvement of tics, comorbidities, and quality of life was demonstrated, while single patients benefitted continuously from thalamic DBS. In another RCT (using the CM-Voi of the thalamus), six patients were included and a significant tic reduction of 37% was described in the blinded phase (*p* = 0.046) and of 49% (*p* = 0.028) after one year of open label phase [31]. Finally, in 2021 Baldermann et al. [32] published results of their RCT in which eight patients with TS were included. The authors investigated the course of tic severity, comorbidities and quality of life during thalamic stimulation (Cm-Voi). The patients were assessed at baseline, after 6 months and 12 months after the surgery in the open label phase. Double-blind phase consisted of sham-controlled periods that took place at 6 and 12 months after the implantation and the patients received 48 h ongoing DBS (ON) followed by 48 h with sham stimulation (OFF) or vice versa in a crossover design. In double blind phase, significant tic reduction according to YGTSS (*p* = 0.001) occurred. In particular, the YGTSS tic scores were significantly decreased with active stimulation by 26% compared to discontinued stimulation after 6 months and by 44% after 12 months. In open label phase YGTSS tic scores decreased significantly from baseline to 6 months (*p* < 0.001) and 12 months (*p* = 0.001) but not from 6 to 12 months (*p* = 1.0).

With respect to psychiatric comorbidity, only limited information is available. In 6 of 8 RCTs [25–27, 29, 30, 32], the effects of DBS (at different targets) on psychiatric symptoms are reported suggesting that DBS may also have beneficial effects on depression and/or anxiety. Effects on OCS and OCD varied, while there seems to be no effect on ADHD (for details see Table 3). It can be speculated that beneficial effects on depression and anxiety might be—at least in part—secondary due to improved tics. Furthermore, it has been speculated that assessment scales used for psychiatric symptoms might be less appropriate for use in patients with TS [31].

**Table 3:** Effect of DBS on psychiatric comorbidities in TS

Authors	Study type	N*	Comorbidities assessed	Results of the double blind phase	Results of the open label phase
Houeto et al. [25]	RCT	1/1	Depression, anxiety, impulsivity	Range of change of − 8 to 60% in depression (MADRS) Range of change of 0 to + 110% in anxiety (BAI) Range of change of –14 to 55% in Impulsivity (BIS) Reduction of self-injurious behavior	Improvement of self-injurious behavior
Maciunas et al. [26]	RCT	5/5	Depression, anxiety, OCD	Trends towards improvements in depression (BDI, HAM-D), OCD (Y-BOCS), and anxiety (HAM-A)	A non-significant trend for decreased verbal fluency, memory, and sustained attention and reaction time at the 3-month follow-up compared with levels at the preoperative assessment BDI-2,HAM-D, HAM-A, and Y-BOCS showed a trend toward improved mood, reduced anxiety, and fewer obsessions and compulsions
Welter et al. [27]	RCT	3/3	Depression, anxiety, OCD	Improvement during both pallidal and thalamic stimulation in 2/2 patient with depression (MADRS) Improvement of anxiety in 2/2 patients with anxiety (BAI) No change in OCD (0/0) (no scale used)	A dramatic reduction in self-injurious behavior and impulsiveness
Ackermans et al. [31]	RCT	6/6	Depression, anxiety, OCD, ADHD, self-injurious behaviors	No effect on any comorbidities (Y-BOCS, CAARS, BAI, BDI)	No effect on any comorbidities (Y-BOCS, CAARS, BAI, BDI)
Kefalopoulou et al. [30]	RCT	13/15	Depression, anxiety, OCD	No improvement of depression (*p* = 0.127, BDI) No improvement of anxiety (*p* = 0.352, STAI) No improvement of OCD (*p* = 0.979, Y-BOCS)	Significant improvement of mood (BDI) Modest, non-significant effects in OCD (Y-BOCS) and anxiety (STAI)
Welter et al. [29]	RCT	16/16	Depression, anxiety, OCD	No improvement of depression (*p* = 0.25, MADRS and *p* = 0.08, HAM-D) No improvement of OCD (*p* = 0.25, Y-BOCS) No improvement of anxiety (*p* = 0.91, BAS)	Significant changes between inclusion and the end of the open-label period in anxiety (MADRS, HADS) No significant changes in depression (BAS, HADS) and OCD (Y-BOCS)
Müller-Vahl et al. [28]	RCT	10/10	OCD, ADHD, depression, anxiety	No effect on any comorbidities (Y-BOCS, CAARS, BAI, BDI)	OCD completely remitted (Y-BOCS) None of the patients exhibited a reduction in CAARS values of ≥ 30% at any follow-up visit Due to incompliance, only incomplete data were available without convincing evidence suggesting changes in mood No changes in anxiety (STAI)
Baldermann et al. [32]	RCT	8/8	Depression, OCD, anxiety, ADHD	Not assessed	Significant improvement of OCD, depression and anxiety
Baldermann et al. [36]**	M	156	Depression, OCD	Median reduction of 31.25% in OCD (Y-BOCS) Median reduction of 38.89% in depression (BDI)	Median reduction of 31.25% in OCD (Y-BOCS) Median reduction of 38.89% in depression (BDI)
Coulombe et al. [35]**	M	58	Depression, anxiety, OCD	Range of change of − 75 to + 100% in OCD (Y-BOCS) Range of change of − 2.6 to + 58% in anxiety (STAI) Range of change of − 80 to + 100% in depression (HAM-D)	Range of change of − 75 to + 100% in OCD (Y-BOCS) Range of change of − 2.6 to + 58% in anxiety (STAI) Range of change of − 80 to + 100% in depression (HAM-D)

Included are all RCTs and meta-analyses giving respect data

*DBS* deep brain stimulation, *TS* Tourette syndrome, *M* meta-analysis, *RCT* randomized controlled trial, *OCD* obsessive–compulsive disorder, *ADHD* attention deficit hyperactivity disorder, *Y-BOCS* Yale-Brown Obsessive–Compulsive Scale, *BDI* Beck Depression Inventory, *STAI* The *State-Trait Anxiety Inventory*, *HAM-A* Hamilton Anxiety Rating Scale, *HAM-D* Hamilton Depression Rating Scale, *MADRS* the Montgomery–Åsberg Depression Rating Scale, *BAI* Beck Anxiety Inventory, *BIS* the Barratt Impulsiveness Scale, *CAARS* the Conners’ Adult ADHD Rating Scales

*The first number refers to the number of patients, included in the double- blind controlled study phase, the second number refers to those in the open uncontrolled study phase, **this study did not distinguish between RCT and open studies. No data are included from the International Tourette DBS Registry and Database, since no results on comorbidities were given

### The International Deep Brain Stimulation Database and Registry

In 2018, data from a large international cohort were published based on the International Deep Brain Stimulation Database and Registry [33]. At the time, this registry included 185 patients with otherwise treatment-refractory TS, who underwent DBS implantation between 2012 and December 2016 at 31 institutions in 10 different countries (exact details about methodology of the data collection and analysis can be consulted in the paper by Martinez-Ramirez et al. [33]). The authors mainly focused on the efficacy of DBS in reducing tics at 6 and 12 months after DBS implantation as measured using the YGTSS-TTS) and the number and profile of adverse events (AEs) related to both surgery (such as infections and hemorrhage) and stimulation (such as paresthesias, bradykinesia, depression, and dystonia). In addition, sub-analyses were performed with respect to the target used [33]. The study reports that: (i) on average, DBS resulted in a tic improvement of 45.1% according to the YGTSS-TTS, (ii) the overall rate of AEs was high (35.4%) and most of the AEs were stimulation-related (30.8%), whereas 3.8% were surgery-related and only 1.3% were device-related, (iii) the most frequently used target was the centro-median thalamic region (57.1%, 93/163 patients), followed by the anteromedial part of the GPi (25.2%, 41/163 patients), the postero-ventrolateral part of the GPi (15.3%, 25/163 patients), and the anterior limb of the internal capsule (ALIC) (2.5%, 4/163 patients). Detailed data on the AEs are shown in subsequent paragraphs. Data obtained from other stimulation targets were not given. There was no evidence for superiority of a particular target [33]. Data from the same registry were complemented by a follow-up analysis published in 2019 [34]. Details are presented in the “Retrospective analysis on long-term follow-up” section.

### Meta-analyses

To date, two meta-analyses have been published investigating efficacy and safety of DBS in adults (published in 2016) [36] and in children and youth [35] (published in 2018). Baldermann et al. [36] included 57 studies (both controlled and uncontrolled) with a total of 156 adult patients. Based on these data, DBS resulted in an average improvement of tics of 52.7% on the YGTSS-TTS (IQR = 40.74, *p* < 0.001). Analysis of data from controlled studies only (four studies with a total of 27 patients [25, 26, 30, 31]) favored “on stimulation” versus “sham stimulation” with a standardized mean difference of 0.96 (95% CI 0.36–1.56).

With respect to the target (thalamus, posteroventrolateral part of the GPi, anteromedial part of the GPi, ALIC, and nucleus accumbens (NA), no significant differences were found in tic reduction, when analyzing data of all 57 studies together [36]. However, further analyses suggested that different patient groups benefitted differently from stimulation at different targets. In particular, thalamic stimulation was more effective when tics were less severe. In contrast, YGTSS-TTS after GPi DBS correlated positively with preoperative impairment score of the YGTSS, but not with the tic specific score (YGTSS-TTS). Regarding psychiatric comorbidities, Baldermann et al. [36] found a median reduction of OCD of 31.3% on the Yale-Brown Obsessive Compulsive Scale (Y-BOCS). Subgroup analysis did not show a difference in reduction of OCD symptoms between targets (*p* = 0.812). Moreover, there was a median improvement of mood of 38.9% (measured with the Beck Depression Inventory (BDI)). Again, subgroup analysis did not show differences between targets (*p* = 0.692).

In the second meta-analysis data on efficacy and safety of DBS specifically in children and youth (aged 12–21 years, mean age 17.9 ± 2.7) is summarized based on 21 studies including 58 cases. The authors report an average tic improvement of 57.5% ± 24.6% [35]. Comorbid depression correlated negatively with outcome (*p* < 0.05). In patients with less severe tics greater improvements were evident following thalamic stimulation. More than one-quarter (*n* = 16, 27.6%) of participants experienced AEs, mostly mild in severity. For further details please consult the subsequent section dedicated to AEs. The authors’ interpretation of the data is that in carefully selected children and youth with treatment refractory TS, DBS is an effective treatment for tics with a moderate safety profile. In none of the meta-analyses, any predictors were found that allow a prognosis of outcome after DBS.

### Retrospective analysis on long-term follow-up

In 2019, Johnson et al. [34] published results of the largest retrospective analysis so far based on the International Deep Brain Stimulation Database and Registry involving 13 sites from North America (39 patients), Europe (63 patients) and Asia (21 patients). They assessed the effects of DBS in the long-term follow-up (up to 96 months) of 110 patients with TS, who were implanted in the CM-Pf and the CM/Voi of the thalamus (*n* = 51), the GPi (*n* = 47), the NA/ALIC (*n* = 4) or combinations of these targets (*n* = 8). Similarly to Baldermann et al.’s meta-analysis [36], the authors report that both tics and OCS significantly improved over time (*p* < 0.01). The median time to reach a 40% improvement of tics was 13 months. However, no significant differences were found between different brain targets (*p* > 0.05). Just recently, Kimura et al. [96] reported about findings from clinical practice and outcome of DBS in TS in Japan. They included 25 patients with refractory TS treated with thalamic CM-Pf DBS. Compared to baseline, tic severity measured by YGTSS-TTS improved by 45.2% at 1 year, and by 56.6% at last follow-up 3 years after surgery. Besides reduction of both motor and vocal tics, an improvement of quality of life was observed.

### Target selection

To date it is not clear which target should be selected in TS. The most often used targets are different parts of the thalamus (CM–Pf and CM/Voi) and the postero-ventrolateral and the anteromedial part of the GPi. However, several other targets have been suggested for the management of tics (and comorbidities) in TS including the NA, the ALIC, the globus pallidus externus (GPe), the subthalamic nucleus (STN) and the H Fields of Forel.

According to both the International Deep Brain Stimulation Database and Registry and the two meta-analyses [35, 36], no significant differences between targets could be detected. However, both analyses reported that stimulation at the anteromedial GPi resulted in slightly, but non-significantly higher improvement rates compared to thalamic (centromedian region) DBS—followed by the postero-ventrolateral GPi.

Until today, only three small controlled trials (including 1, 3 and 10 patients, respectively) directly compared two different targets within patients with TS [25, 27, 28]. Houeto et al. [25] (*n* = 1) found that stimulation of either target improved tic severity by 70%, markedly ameliorated coprolalia, and eliminated SIB. Welter et al. [27] (*n* = 3) reported that GPi stimulation resulted in better tic control compared to thalamic DBS, while simultaneous stimulation at both targets did not result in further improvement. However, only thalamic DBS had a positive effect on depressive mood, emotional hypersensitivity, anxiety, and impulsiveness. Another group also reported about increasing incidence of side effects and decreasing efficacy at long-term follow-up in seven patients after thalamic DBS [37]. Moreover, beneficial effects on tics and global functioning of stimulation of unilateral pallidal and nigral thalamic territories have been reported in two patients who presented predominantly with one-sided (contralateral) tics [123]. Finally, a recently published RCT [28] compared efficacy and safety of bilateral thalamus (CM-Voi) versus pvl GPi versus sham stimulation in severe medically refractory GTS. After 36 weeks GPi DBS—but not thalamic DBS resulted in a significant tic reduction compared to baseline, but had no effect on premonitory urges and psychiatric comorbidities. Direct comparisons of both targets to sham stimulation resulted in inconsistent or negative findings. At long-term follow-up on average 6 years after surgery, at group level no significant improvements could be detected, although single patients continuously benefitted from thalamic DBS. Just recently, Servello et al. [124] published results of a retrospective study comparing effects of CM-Voi (*n* = 41) to antero-medial GPi (*n* = 14) stimulation. During an evaluation period of 48 months, both targets were equally effective in reducing tics and beneficial effects persisted over time. With respect to OCD, GPi DBS was superior compared to thalamic stimulation.

Based on available data, it is conceivable that in TS, different targets are comparably effective as is the case in DBS in Parkinson’s disease. Whether the GPi and thalamic nuclei form an interconnected common network in terms of a network-modulation involved in symptom improvement of TS is currently under investigation. From preliminary experimental studies a new approach has been suggested by selecting individual targets based on the identification of patterns of connectivity [38, 71, 125].

### Adverse events

When evaluating the value of DBS in TS, type and frequency of AEs has to be considered too. According to the International Deep Brain Stimulation Database and Registry [33], more than one third of patients (35.4%) experienced AEs. The most frequently reported AEs were stimulation-related (30.8%) and included dysarthria and paresthesias. Dystonia and dyskinesias were more frequently reported after GPi DBS, while paresthesias and weight gain were more frequently reported after thalamic DBS. The only surgery-related AEs (in 3.8%) were hemorrhage found in 1.3% of cases and infections, which occurred with a rate of 2.5%. Only 1.3% of AEs were device-related. Overall, in TS the average rate of AEs after DBS seems to be similar compared to other patient populations such as dystonia and Parkinson’s disease [39, 126, 127].

In contrast to the data from the International Deep Brain Stimulation Database and Registry [33], but in line with other studies [24, 25], Servello et al. [72] reported a higher risk of infections in patients with TS (18%, *p* < 0.001) compared to other populations. In a recently published meta-analysis in different populations [127], a mean prevalence rate of surgical site infections of 5.0% was reported. Infection rates above average were found in epilepsy (9.5%), dystonia (6.5%), and TS (5.9%), while rates were lower in OCD (4.5%), Parkinson’s disease (3.3%), essential tremor (2.9%), and multiple sclerosis (2.4%). It has been speculated that increased infection rate in TS might be related to complications caused by SIB or alternatively to an underlying immunological dysfunction related to the pathology of TS [36]. However, in a recent study based on the cohort of the Tourette Association of America’s International Tourette Syndrome Registry and Database [128], the incidence of lead removal was only 5.6% and hence lower than previously reported rates in TS [72]. Infections accounted for nearly half of DBS explantations in this cohort. Partly contradictory data might be explained by lack of harmonized methodology to assess AEs such as a unified questionnaire. Further details of the AE profile reported in RCTs and meta-analyses are given in Table 4.

**Table 4:** Adverse effects of DBS in TS

Study	Type of the study	Number of patients included	Total number of different AEs	Stimulation-related AEs*	Procedure-related AEs*	Absolute number of infections
Baldermann et al. [36]	M	156	Not reported	Number of events not reported, descriptive results: gaze disturbances, mood deterioration, dysarthria, psychotic symptoms, erectile dysfunction, memory impairment, anxiety, weight gain, agitation, tiredness, nausea, hypotonia, impulsivity, dizziness, poor balance, speech problems, worsening of tics, hypomania, suicide attempt, apathy	Not reported	Not reported
Coulombe et al. [35]	M	58	16	*N* = 16, tension headache, worsening of preexisting tremor, transient blurring of vision, dizziness, decreased memory, seizure-like episode, suicidal thoughts, decline of attention and mental flexibility, neck tightness, paraesthesias, headedness, parkinsonism, increased OCD, anxiety, agitation, disturbance of eye mobility, dysarthria, nausea, lead removal	*N* = 8, infection, hematoma, wound revision, subcutaneous hydrops, hardware malfunction, lead tip cyst, lead fracture	*N* = 3
Houeto et al. [25]	RCT	1/1*	5	*N* = 5, paraesthesias in the contralateral half of the tongue, contraction of the contralateral half of the body, nausea, hypotonia, and anxiety	*N* = 0	*N* = 0
Maciunas et al. [26]	RCT	5/5*	4	*N* = 2, acute psychosis, recurrence of tics	*N* = 0	*N* = 0
Welter et al. [27]	RCT	3/3*	6	*N* = 6, lethargy, transientcheiro-oral or arm paresthesias, nausea, vertigo, anxiety, libido decrease	*N* = 0	*N* = 0
Ackermans et al. [31]	RCT	6/6*	9	*N* = 5, lack of energy, nystagmus, blurred vision, fixation problem, impaired vertical gaze	*N* = 3, parenchymal hemorrhage, infection, varying motor and psychiatric symptoms (lethargy, binge eating, dysarthria, apathy, gait disturbances and frequent falls)	N = 1
Kefalopoulou et al. [30]	RCT	13/15*	23	*N* = 19, deterioration of condition, hypomania, increased anxiety, insomnia, irritability, tiredness, headaches, mood deterioration, emotional lability, upper-respiratory tract infection, abdominal rush, panic attacks, mild dysarthria, lower limb dyskinesia	*N* = 7, battery infection, prolonged pain around IPG, keloid scar, burr-hole cap discomfort, connection cable discomfort, upper-respiratory tract infection	*N* = 2
Welter et al. [29]	RCT	16/16*	29	*N* = 15, increase in tic severity and anxiety, depression, transient loss of balance, nausea and vertigo, sleep disorder, falls, dysarthria, abnormal movements resembling dyskinesia, weight gain	*N* = 14, infection leading to removal of stimulator and electrodes, misplacement of electrode, serious suffusion around head scar, device migration, headache, asthenia, hardware-related pain,	*N* = 6
Müller-Vahl et al. [28]	RCT	10/10*	20/16	*N* = 31, headache, dizziness, paraesthesia, teeth gnashing, tremor of extremities, tiredness, double vision, dystonic movements of the hand, speech blockade, increase of tics, dysphoria, sleeping problems, deterioration of OCD	N = 5, cable dysfunction, superficial wound infection, chronic infections of stimulator’s area (subclavical region)	N = 4
Baldermann et al. [32]	RCT	8/8	12	*N* = 6, Increase in self-injurious behaviors, increased irritability, dysarthria, micrographia	*N* = 2, postoperatively measured increased impedances, discomfort with cable	*N* = 0

*DBS* deep brain stimulation, *TS* Tourette syndrome, *M* meta-analysis, *RCT* randomized controlled study, *AEs* adverse event(s)

*Given is the total number and in addition a description of each AE

## Recommendations from recently published guidelines

Since the publication of the first ESSTS DBS guidelines in 2011, further guidelines/recommendations specifically for DBS in patients with TS have been published: (i) in 2015 by the Tourette Syndrome Association International Deep Brain Stimulation (DBS) Database and Registry Study Group [129], (ii) in 2019 by an international team of the American Academy of Neurology (AAN) [5], and (iii) in 2021 by an international group of experts [130]. In addition, in 2014 members of different psychiatric and neurosurgical societies published more general consensus guidelines on DBS in psychiatric disorders [131]. Conclusions from the systematic review of the AAN [5] were that (i) the optimal brain target for DBS in TS is still unknown, (ii) DBS of the anteromedial GPi seems to be more effective in reducing tics than sham stimulation, (iii) there is insufficient evidence to confirm efficacy of DBS of thalamic nuclei, and (iv) AEs including infection and removal of hardware, appear more common in patients with TS compared to patients receiving DBS for other indications. However, AAN recommendations [5]—as well as the consensus guidelines published in 2014 [131]—deserve some comment regarding the target selection. In both guidelines, an RCT published by Ackermans et al. [31] describing the results of thalamic DBS in TS was not considered, presumably because of the small sample size of only six patients.

The International Deep Brain Stimulation Database and Registry Study Group [129] gave the following recommendations: (i) patients qualified for the procedure should have a diagnosis of TS with severe motor and vocal tics, which did not respond to behavioral interventions per current expert standards and pharmacological treatments from three pharmacological classes (including alpha-adrenergic agonist, two dopamine antagonists (typical and atypical), and a drug from at least one additional class (e.g., clonazepam, topiramate, tetrabenazine)), (ii) DBS should be offered only to patients in centers that have experience with DBS for this indication and after critical evaluation by a multidisciplinary team, (iii) tics as well as psychiatric comorbidities should be assessed and quantified rigorously pre- and post-operatively, and (iv) functional tics and malingering should be considered in the differential diagnosis.

In 2021, Martino et al. [130] suggested the following principles of DBS in TS: (i) tics should be defined as harmful or malignant for a minimum period of 6 months or should be scored as ≥ 35 on the YGTSS-TTS for a minimal period of 12 months, (ii) the patient should report at least moderate impairment on the YGTSS impairment score or high impairment on the Gilles de la Tourette Quality of Life Scale (GTS-QoL) for a minimal period of 12 months, (iii) patients should be treatment resistant defined as no response to behavioral and/or pharmacological interventions, (iv) co-existing psychiatric conditions should be stable for a minimal period of 6 months prior to surgery and primary impairment of quality of life should be caused by tics, and (v) patients should be older than 18, but in severe cases, patients younger than 18 can be considered.

Since the introduction of DBS in TS, recommendations regarding a minimum age for DBS surgery changed substantially. In 2006, the first guidelines of the Tourette Association of America (TAA) proposed a minimum age of 25 years to ensure that individuals would be past the age of potential spontaneous tic improvement as part of the natural course of the disease, before they were implanted with a surgical device [137]. However, more recent guidelines do not recommend such a rigid age limit any more [5, 129, 130]. Compelling arguments have been made for surgical intervention at younger ages in certain cases of severe TS, given the impact extreme tics can have on the emotional developmental, education, professional development, and relationships. However, some expert groups recommend to involve the local ethics committee in patients under the age of 18 before surgery. Similar suggestions have been made in patients where DBS is considered as “urgent” [129, 130, 138].

In summary, recommendations given in the previously mentioned guidelines are (unsurprisingly) largely overlapping with respect to (i) the involvement of a multidisciplinary team consisting of a psychiatrist or neurologist, neurosurgeon, and neuropsychologist, (ii) confirmation of the diagnosis with severe and/or self-injurious tics that are medication- and behavioral therapy-refractory, and (iii) adequate assessment and treatment of psychiatric comorbidities.

## Results of the ESSTS survey

Our recent online survey among a large number of ESSTS members (*n* = 59) from 17 different European countries addressed specific questions regarding DBS in TS. Although DBS is offered to patients with TS in the particular region (49%) and center (25%), respectively, and DBS is accessible in the context of both clinical practice (in 29% of centers) and of clinical trials (in 15%), surgery is actually performed only rarely. ESSTS experts estimated to consider DBS in only about 2.5% (mean, ± 5.2% (SD)) of their patients. On average, only 2.1 (± 5.7) patients per center recommended for DBS underwent surgery in the past 5 years. There is broad consensus among ESSTS experts to consider DBS only in carefully selected, severely affected and otherwise treatment-refractory patients. Only two experts (3%) reported to consider DBS “routinely, if behavioral and medical (at least three different drugs) treatment failed to improve tics or caused significant AEs”, while 17% indicated to consider DBS “not at all/never”. The majority of ESSTS members (59%) reported to consider DBS also in patients under the age of 18 years. Remarkably, patients’ interest in DBS seems to be low: according to experts’ judgement even in specialized centers on average less than two patients/year (mean = 1.8 ± 2.7) ask for DBS.

Although results obtained from the ESSTS survey are affected by several limitations (e.g. small number of participants, no data on how many patients are seen/center relative to those numbers of patients, who were proposed for DBS and who finally underwent surgery in this specific center). However, results from the survey are completely in line with clinical experience in large European TS centers demonstrating that (i) only very few patients ask for DBS, (ii) DBS is recommended only to a very small number of patients, and (iii) not all patients decide for DBS although recommended by an expert.

## Future developments

As a new development in conventional DBS, a new approach is suggested for stimulation of patients with various movement disorders including TS using closed-loop adaptive DBS (aDBS) [139]. With this strategy pathological patterns of neuronal activity are identified [140] allowing flexible dynamic adaptation of stimulation parameters fine-tuned to the concurrent therapeutic demand. This approach might help to decrease the incidence of stimulation-related AEs and to preserve the battery for a longer period of time [97, 141]. A requirement for the development of closed loop DBS for TS is the identification of neurophysiological or clinical markers of tic activity. Recently tic-dependent transient rate changes were found in the activity of individual neurons of the anterior (associative/limbic) GPe and GPi of 8 awake patients during DBS electrode implantation surgeries [98]. Finally, use of tractography may be helpful to optimize the technique, to individualize the targeting, and to increase DBS efficacy [99]. Future developments should focus on the application of these novel techniques to identify non-responders as up to this day it is not clear which parameters predict response to DBS.

## Updated recommendations of the ESSTS DBS guidelines group

Currently no definite conclusions can be drawn on efficacy and safety of DBS in TS and many issues are still a matter of debate (Table 5). Therefore, surgical treatment should still be considered as an experimental therapeutic option for carefully selected patients with otherwise treatment-refractory tics. Noteworthy, RCTs consistently reported fewer positive effects on both tics and psychiatric comorbidities compared to data obtained from uncontrolled studies. This fact should also be taken into consideration when interpreting the results from the available data, meta-analyses, and from the International Deep Brain Stimulation Database and Registry, since the majority of these results are based on open and uncontrolled studies. Furthermore, there are specific DBS-related methodological difficulties that may hamper RCTs (e.g., very limited study population meeting inclusion criteria, fixed stimulation parameters vs. individually adapted stimulation parameters, inability of adequate blinding, and handling very severely affected subjects who may suffer from SIB in a clinical trial). Finally, DBS RCTs in psychiatric disorders more often fail to show positive results, or show minor results because of the limited time frame, as in the case of (ALIC) DBS RCTs for OCD [142], and depression [143, 144].

**Table 5:** Issues on DBS in TS worth of discussion

Clinical problem	Suggestions	Rationale
Age limit	No age limit is recommended	In 2006, an expert group [132] recommended an age limit of 25 years (with rare potential exceptions). However, during the last decades, 58 patients younger than 25 years have received DBS [35]. Since then, different age limits (18 or 21 years) have been suggested by different groups [133]. In 2015, in their updated recommendations Schrock et al. [129] suggested that there should be no age limit, but the decision should be taken individually. Although in the majority of patients tics improve during the course of the disease, it is unclear, whether, when and to what extent severely affected patients may await a significant improvement. Since, there is no evidence that DBS is less efficacious in children, adolescents and young adults with TS—although direct comparisons are missing—evidence is missing justifying any age limit
Functional “tic-like” movement disorder	The diagnosis of a functional “tic-like” movement disorder must be excluded. In particular, co-occurrence of tics and functional “tic-like” movements should be taken into consideration before making the diagnosis of “otherwise treatment-refractory” TS	The diagnosis of a functional movement disorder with “tic-like movements” must be excluded. From clinical experience it can be assumed that the prevalence of functional tic-like movements is increasing and/or physicians are increasingly aware of this phenomenon. Most of these patients present with severe and complex symptoms that do not respond to anti-tic treatments (including antipsychotics and behavioral therapy), and, therefore, might be erroneously misdiagnosed with severe and “treatment-refractory” TS. In addition, there is increasing evidence that in some patients with confirmed TS, in addition, functional tic-like movements coexist. In the literature, this phenomenon has also been described as “tic attacks” [134], since functional tic-like movements may resemble TS-related tic exacerbations. Accordingly, it has been speculated that inconsistent data on DBS in TS might at least in part be influenced by results obtained from patients who suffer—in addition or solely—from functional ‘tic-like’ movements [135, 136]
Primary treatment goal	The reduction of tics—and not the improvement of comorbidities—should be the primary goal of DBS	Only limited data is available regarding efficacy of DBS on psychiatric comorbidities in TS. While there seems to be some beneficial effect on comorbid depression, effects on OCS and OCD varied, and there seems to be no effect on anxiety and ADHD. Therefore, best possible treatment of psychiatric comorbidities should be established prior to surgery
Treatment refractoriness	Most authors suggest to assume ‘‘treatment refractoriness’’, if behavioral interventions as well as pharmacotherapy with 3 different drugs including both a typical and an atypical antipsychotic in adequate dosage over an adequate period of time do not result in a significant tic reduction or lead to unbearable AEs	There is no generally accepted definition available for ‘‘treatment refractoriness’’ in TS. Although different patients may respond in a different way to different antipsychotics, three different drugs seem to be adequate to determine treatment refractoriness. Since clonidine is less commonly used in Europe compared to the USA and many European experts are convinced that clonidine is only effective in case of comorbid ADHD and in these cases less effective compared to antipsychotics, we believe that treatment with clonidine must not be undertaken to determine ‘‘treatment refractoriness’’. Although several other drugs have been recommended for the treatment of tics, in Europe none of these drugs is approved or can be recommended without reservation. Although haloperidol is the only drug that is formally licensed in many European countries for the indication tics and TS, due to relevant AEs it can no longer be recommended without restrictions. Since treatment strategies also depend on availability and approval in respective countries, we recommend not to stick to a specific number of different treatments before DBS, but suggest to use at least three different drugs before considering DBS
Target	Altogether eight different targets have been suggested for DBS in TS. Based on current knowledge most experts recommend to use either thalamus (CM–Pf and/or CM–Pf/Voi) or GPi (postero-ventrolateral or anteromedial)	Currently, there are no generally accepted predictors known suggesting superiority of one target over another one. Recommendation for use of thalamus and GPi DBS is largely based on the fact that these targets have been used much more common compared to all other targets. It has been speculated that different targets might be comparable effective, since they may form a common network involved in TS
Number of electrodes and targets	In the vast majority of patients, bilateral stimulation at one target has been performed. Only in single cases unilateral DBS at one target or simultaneous stimulation at two targets has been performed.	Bilateral stimulation at one target is the standard procedure in TS. However, simultaneous implantation of 4 electrodes at two targets has alternatively been suggested to increase options for stimulation without further surgery. If tics mainly occur on one body part, (contralateral) unilateral DBS can be taken into consideration

*DBS* deep brain stimulation, *TS* Tourette syndrome, *YGTSS* Yale Global Tic Severity Scale, *AEs* adverse events, *GPi* globus pallidus internus, *CM-Pf* centromedian parafascicular, *OCD* obsessive–compulsive disorder, *OCS* obsessive–compulsive symptoms, *ADHD* attention deficit hyperactivity disorder, *AEs* adverse events

The ESSTS DBS guidelines group has the following recommendations for DBS in TS:

1. A primary diagnosis of TS according to DSM-5 or ICD-10 criteria must be confirmed. These guidelines do not apply to treatment of patients with secondary tics due to other neurological diseases.
2. The diagnosis of a functional “tic-like” movement disorder must be excluded. In particular, co-occurrence of tics and functional “tic-like” movements should be taken into consideration before making the diagnosis of otherwise treatment-refractory TS [135].
3. The reduction of tics—and not the improvement of comorbidities—should be the primary goal of DBS in patients with TS [145]. Adequate treatment of psychiatric comorbidities should be established prior to surgery.
4. DBS should be taken into consideration only in patients with tics that cause significant impairment in patients’ quality of life and that are resistant to established conservative treatment strategies including behavioral and pharmacotherapy. Because (i) treatment strategies for tics differ from country to country, (ii) treatment strategies are based on availability of therapies, and (iii) an established definition for “treatment refractoriness” is still lacking, the working group decided not to recommend a specific treatment algorithm before considering DBS. However, before undergoing DBS the patient should be regarded as “otherwise treatment-refractory” in the opinion of a multidisciplinary team which includes refractoriness to both behavioral and pharmacotherapeutical interventions [131].
5. Both expected treatment efficacy and tolerability must be taken into consideration before the decision on DBS is taken.
6. We do not recommend a minimum age for operation. However, physicians should be aware that (i) tics often improve spontaneously after puberty and in early adulthood, (ii) the typically spontaneous improvement of tics with increasing age should be thoroughly discussed, (iii) in children—as in adults—abrupt and tremendous deterioration of symptoms might be related to the onset of comorbid functional “tic-like” movements [134] and not as a result of an increase of tics caused by TS, (iv) there is only very limited information on the efficacy of DBS in children with TS, and (v) in children and adolescents involvement of the local ethics committee is recommended.
7. DBS should be performed in specialized centers by a dedicated multidisciplinary team including a psychiatrist, psychologist and/or neurologist trained in the treatment of patients with TS. This multidisciplinary approach is not only required preoperatively (e.g. for correct diagnosing and assessment of inclusion criteria and judgement on “treatment-refractoriness”), but also during follow-up treatment with regular consultations of both surgical as well as psychiatric or neurological experts.
8. If possible, DBS should be performed following a specified protocol and within the context of a controlled trial, a cohort study, or registry database, but at least should include systematic pre- and post-DBS assessment of tics, premonitory urges, psychiatric comorbidities (including at least ADHD, OCD, depression, and anxiety), and quality of life.
9. Physicians should be aware and take into consideration that AEs—and in particular infections—might be more common in patients with TS.
10. Since it is still unclear, which target is most effective in TS in general and specifically for tic reduction, we do not recommend using a particular target. Based on current data different parts of the thalamus (CM–Pf and CM/Voi) as well as the postero-ventrolateral and the anteromedial part of the GPi seem to generate comparable results. Long-term follow-up of patient characteristics is essential and systematic documentation of long-term outcomes may help disentangle whether specific features of TS are more likely to be responsive to thalamic or pallidal DBS.

## Data Availability

Not applicable.
